# Recurrent syncope driven by unique‐variant angina pectoris

**DOI:** 10.1002/ccr3.8460

**Published:** 2024-02-01

**Authors:** Guang Yang, Yanna Lei, Minglong Xin, Wenhu Xu, Guangxian Zhao, Xueying Jin, Meina Piao, Xiang Li, Xian Wu Cheng

**Affiliations:** ^1^ Department of Cardiology and Hypertension, Jilin Provincial Key Laboratory of Stress and Cardiovascular Disease Yanbian University Hospital Yanji Jilin China; ^2^ Key Laboratory of Natural Medicines of the Changbai Mountain, Ministry of Education Yanbian University Yanji Jilin China

**Keywords:** case report, coronary artery spasm, syncope, variant angina

## Abstract

The patient's vasospastic variant angina manifested as syncope with asymptomatic ischemic episodes, and repeated 24‐h dynamic electrocardiogram and coronary angiography examinations combined with coronary provocation spasm tests were necessary for its diagnosis and management.

## 1 INTRODUCTION

Vasospastic angina (VSA) is variant form of angina pectoris that often occurs at night or at rest, with transient ST‐segment elevation observed on electrocardiography (ECG) during an attack of chest pain.^1^ Cardiogenic syncope is often due to severe arrhythmias (e.g., high‐degree atrioventricular block, ventricular tachycardia, and ventricular fibrillation) in response to ischemic stress.^2^ Clinical evidence has shown that variant angina manifests as syncope with typical ischemic episodes.^3^ However, some cases of night‐time atypical variant angina‐related cardiogenic syncope are particularly difficult to differentiate from neurogenic syncope, leading to delays in treatment. Lifestyle changes and pharmacotherapy (e.g., non‐dihydropyridines calcium‐channel blockers [CCBs], statins, nitrates, nicorandil, α1‐adrenergic receptor antagonists, rho‐kinase inhibitors, etc.) have been proposed to treat patients with VSA.^4^ Herein, we describe the case of a patient with midnight syncope with no‐flow limiting stenosis; he was eventually observed to have paroxysmal transient ST‐segment elevation with a combined Type II (2:1 downward transmission) and Type I second‐degree AV block that met the diagnostic criteria for vasospastic angina‐induced syncope.

## 2 TIME LINE

The time line of the patient's symptoms and treatment is summarized in Table 1.

**TABLE 1:** The timeline of the patient's symptoms, examinations, treatments, and follow‐up.

Month, day 2020/2023	Chief complaint	ECG	CT (brain) CAG Echocardiogram	SBP/DBP; Troponin I	Treatment
February 25	Emergency medicine with syncope	Not significant	Old multiple lacunar infarctions	152/101 mmHg; Normal	Telmisartan 40 mg/d
March 12	Emergency medicine with syncope	Unclear	–	165/100 mmHg; Normal	Telmisartan 40 mg/d Compliance is poor
April 21	Emergency medicine with syncope	24‐h dynamic ECG: sinus rhythm and atrial premature beats	Old multiple lacunar infarctions	155/96 mmHg; Normal	Telmisartan 40 mg/d Compliance is poor
April 22	Admission to cardiology Department	–	–	145/95 mmHg; Normal	Aspirin 100 mg/d Ticagrelor 90 mg/d Atorvastin 10 mg/d Telmisartan 40 mg/d Metoprolol 47.5 mg/d
April 23		24‐h dynamic ECG: sinus rhythm and atrial premature beats	CAG: RCA stenosis (<50%)	–	No change
April 25		24‐h dynamic ECG: sinus rhythm and atrial premature beats	LAD 28 mm; IVST/LVPWT 10/15 mm; LVDd/LVSd 25/45 mm; LVEF 60%; FS 39%.	140/90 mmHg; –	No change
April 27		24‐h dynamic ECG: ST‐segment elevated 0.1–0.2 mV in the II, III, and avF leads	–	138/88 mmHg; Normal	Lifestyle intervention (e.g., reducing salt intake, prohibiting smoking, etc.) Atorvastin 10 mg/d Telmisartan 80 mg/d Diltiazem 30 mg, 3×/d
April 29	Discharge	–	–	–	No change
July 30	Cardiology outpatient: Variant angina (−)/syncope (−)	Normal	–	126/84 mmHg	Lifestyle intervention Aspirin 100 mg/d Atorvastin 10 mg/d Amlodipine/Losartan 1 tablet/d
Oct. 29	Telephone interview: Variant angina (−)/syncope (−)	–	–	–	No change
April 30, 2021	Cardiology outpatient Variant angina (−)/syncope (−)	ECG: not significant	–	128/84 mmHg	No change
May 10 2022	Telephone interview: Variant angina (−)/syncope (−)	–	–	–	No change
May 22 2023	Cardiology outpatient Variant angina (−)/syncope (−)	24‐h dynamic ECG: Sinus rhythm Frequent atrial premature beats and short‐term atrial tachycardia	Echocardiogram: LAD 31 mm; IVST/LVPWT 8/9 mm; LVDd 43 mm; LVSd 26 mm; LVEF 64%; FS35%	126/80 mmHg; Normal	No change

Abbreviations: CAG, coronary angiography; CT, computed tomography; DBP, diastolic blood pressure; ECG, electrocardiogram; IVST, interventricular septal thickness; LAD, left atrial diameter; LV, left ventricle; LVDd, left ventricular end‐diastolic dimension; LVPWT, left ventricular posterior wall thickness; LVSd, left ventricular end‐systolic dimension; SBP, systolic blood pressure.

## 3 CASE PRESENTATION

A 72‐year‐old Chinese man who experienced syncope three times within 2 months was admitted to our cardiology department. His heart rate was 78 bpm and he was hypertensive (blood pressure 145/95 mmHg). The patient reported a >30‐year regular smoking habit (>8 tobacco cigarettes/day). The laboratory examination revealed that there were no alterations in blood cardiac injury biomarkers with the exception of elevated low‐density lipoprotein cholesterol (4.08 mmol/L; normal range 0.00–3.12 mmol/L) and elevated N‐terminal pronatriuretic peptide (NT‐proBNP, 1455 pg/mL; normal range < 300 pg/mL).

**FIGURE 1:**
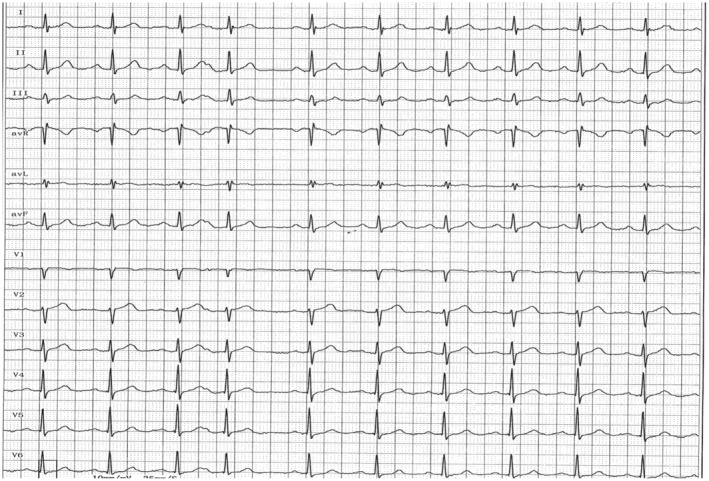
The part of the initial 24‐h dynamic ECG at the time of the patient's presentation without syncope attack. The ECG shows only sinus rhythm and atrial premature beats (101 times), some of which were not downward transmissions.

The initial 24‐h dynamic ECG showed only sinus rhythm and atrial premature beats, some of which were not downward transmissions (Figure 1). The echocardiography data were as follows: left atrium dia., 28 mm; left ventricle (LV) end‐diastolic dia., 44 mm; LV end‐systolic dia., 27 mm; interventricular septum thickness, 10/15 mm; LV end systolic/diastolic diameters 25/45 mm; LV ejection fraction, 60%; fraction shortening, 39%.

**FIGURE 2:**
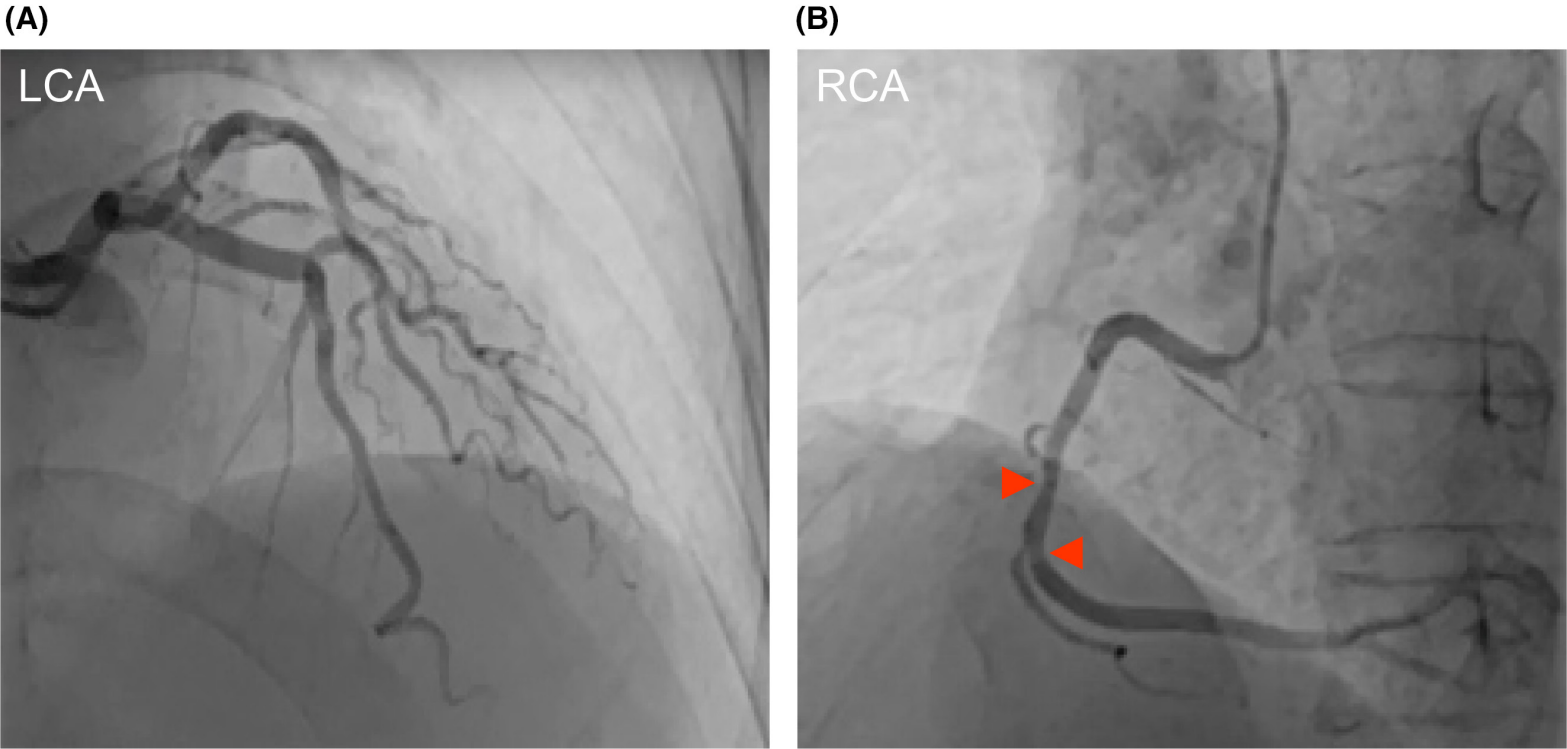
Left coronary artery (LCA) and right coronary artery (RCA). (A) The walls of the left anterior descending and left circumflex arteries were irregular. (B) The middle segment of the RCA with stenosis (<50%).

Coronary angiography (CAG) showed that with the exception of the left main coronary artery, the walls of the left anterior descending and left circumflex arteries were diffuse irregularities (Figure 2A), and the middle segment of the right coronary artery (RCA) showed <50% stenosis (Figure 2B). Color Doppler ultrasound of the carotid and vertebral arteries demonstrated that the intima‐media thickness of the bilateral carotid artery was thickened with multiple plaques.

A non‐dihydropyridine CCB, that is, diltiazem, combined with lifestyle interventions and intensive antilipid and antihypertension medication prevented cardiac ischemic and syncope episodes for 36 months. The patient's treatments are provided in Table 1.

This case presented several characteristics: (1) all of the symptoms (syncope) occurred in the period from midnight to early morning, and the patient reported no fatigue or emotional excitement. (2) The sign of angina pectoris was not chest tightness/typical pain, but a feeling of needing to defecate. (3) The patient's ECG findings were normal in the general examination, and we eventually observed a transient ST segment elevation with a combined Type II (2:1 downward transmission) and Type I second‐degree AV block. (4) CAG showed only mild lesions in the RCA. (5) The patient was an elderly male smoker with hypertension and dyslipidemia, all of which are independent risk factors for a coronary artery spasm.

## 4 FOLLOW‐UP

The patient's follow‐up data at 3, 6, 12, 24, and 36 months showed no cardiac ischemic and/or syncope episodes (Table 1).

## 5 DISCUSSION

**FIGURE 3:**
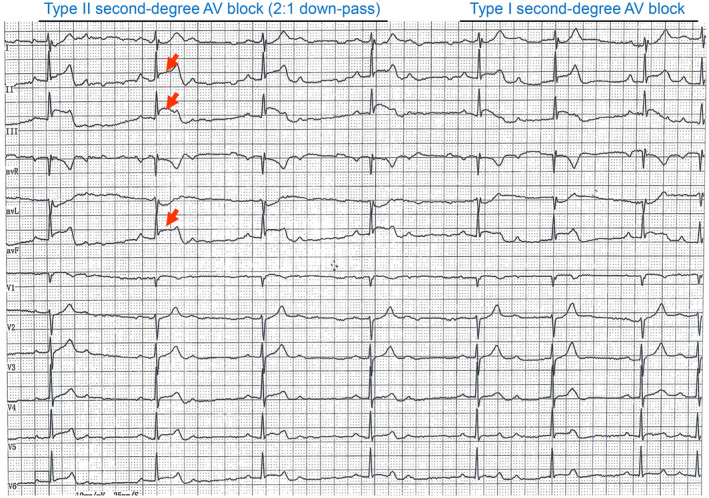
Part of the 24‐h dynamic ECG obtained at the time of the patient's variant angina with syncope attack. ST‐segment 0.1–0.2 mV elevation in the interior leads (II, III, avF) with a combined Type II (2:1 downward transmission) and Type I second‐degree atrioventricular (AV) block.

In this patient's case, the cause of syncope could not be explained by the ECG and CAG results. A fourth 24‐h dynamic ECG screening was thus performed. Fortunately, the ambulatory ECG caught the patient's syncope attack showing sinus rhythm, atrial premature beat (101 times), short atrial tachycardia (one time), and ST‐segment elevated 0.1–0.2 mV in the II, III, and avF leads along with a combined Type II (2:1 downward transmission) and Type I second‐degree atrioventricular (AV) block (Figure 3). These findings suggested that a right coronary spasm may have caused the inferior wall myocardium, AV node ischemia, and severe arrhythmias including the Type II second‐degree AV block, leading to the patient's syncope.^5^

Variant angina refers to a segmental or diffuse reversible spasmodic contraction of coronary artery smooth muscle in the epicardium, which leads to chest tightness, chest pain, arrhythmia, and syncope.^6^, ^7^ In our patient's case, the sign of angina pectoris was not chest tightness/typical pain, but a feeling of needing to defecate. It is rarely seen in cases of variant angina pectoris with syncope as the main manifestation in clinical practice.^4^ The fourth 24‐h dynamic ECG caught the syncope attack and showed a combined Type II (2:1 downward transmission) and Type I second‐degree AV block, and the leads of the ST segment elevation were II, III, and avF (Figure 3), which are lower‐wall leads of the RCA blood‐supplying territory, which is consistent with the CAG finding of mild stenosis in the RCA (Figure 2B). Because 93% of the blood supply of the AV node originates from the RCA,^8^ an RCA spasm appears to have reduced or blocked the blood supply of the AV node in our patient,^5^ leading to the emergence of arrhythmia, including the second‐degree II AV block and/or an even higher‐degree AV block.

In addition, this case of variant angina occurred during the hours from midnight to early morning.^9^ It was reported that during this time of day, the tension of the vagus nerve is increased and vagal excitation affects the conduction system of the heart, aggravating the conduction block.^8^ We thus speculate that our patient's variant angina pectoris manifested mainly as syncope because of the high‐degree AV block caused by a coronary spasm and increased vagal tone. This concept was further supported by our observation that the treatment of the patient with sustained‐release tablets of the non‐dihydropyridines CCB diltiazem— which dilates epicardial and subendocardial coronary arteries and slows down the conduction of the sinoatrial node and atrioventricular node— prevented variant angina and syncope. Moreover, the patient's follow‐up data at 3, 6, 12, 24, and 36 months showed no cardiac ischemic or syncope episodes (Table 1).

Study limitations should be considered. Our patient's case highlights the limitations of routine ECG and CAG for the diagnosis of variant angina pectoris with syncope. It is well known that coronary artery spasm provocation tests (e.g., the acetylcholine challenge test, hyperventilation test, and ergometrine challenge test) can help diagnose variant angina pectoris.^10^ Further research is needed regarding atherosclerotic plaque and vascular spasm in variant angina patients with severe arrhythmia, including those with Type II second‐degree and III AV block, especially regarding underlying coronary artery spasm‐related gene polymorphisms (e.g., endothelial nitric oxide synthase gene),^11^ the clinical progression, and clinical implications.

## 6 CONCLUSION

Twenty‐four‐hour dynamic ECG is an essential tool in the initial screening for variant angina with or without syncope. The important take‐away message of this case report is that repeated 24‐h dynamic ECG examinations will be necessary to rule out unique variant angina (as seen in this rare case) for patients with a high suspicion of cardiogenic syncope and multiple vasospastic factors. In addition, treatment with the non‐dihydropyridine CCB diltiazem combined with lifestyle interventions (smoking cessation and lowered salt intake) and/or intensive lipid reduction (for patients with hyperlipidemia) might be one of the best choices for the management of variant angina patients with or without hypertension. On the other hand, our patient had not only hypertension but also multiple lacunar infarctions with local softening without a fresh cerebral hemorrhage or infarction. He was thus advised to continue taking aspirin. It should be noted that many studies have demonstrated that aspirin is not recommended for patients with VSA without significant organic stenosis.^12^, ^13^, ^14^, ^15^

## AUTHOR CONTRIBUTIONS

**Guang Yang:** Conceptualization; data curation; formal analysis; investigation; methodology; project administration; resources; writing – original draft. **Yanna Lei:** Conceptualization; data curation; formal analysis; supervision; writing – review and editing. **Minglong Xin:** Resources; validation. **Wenhu Xu:** Validation; visualization. **Guangxian Zhao:** Supervision. **Xueying Jin:** Data curation; software. **Meina Piao:** Formal analysis; resources. **Xiang Li:** Conceptualization; data curation; formal analysis; supervision; writing – review and editing. **Xian Wu Cheng:** Conceptualization; project administration; supervision; writing – review and editing.

## FUNDING INFORMATION

This work was supported in part by grants from the National Natural Science Foundation of China (nos. 81,770,485 and 82,370,424 to XWC; no. 82160087 to YL; no. 82060052 to XL).

## CONFLICT OF INTEREST STATEMENT

The authors declare that they have no known competing financial interests.

## CONSENT

The authors obtained the patient's written consent for his case details to be published.

## Data Availability

The data underlying this article will be shared upon reasonable request to the corresponding author.
